# Whom Can I Rely on? The Impact of China's Public Pension Program Expansion on the Expectations for Old-Age Support

**DOI:** 10.1093/geroni/igab046.1376

**Published:** 2021-12-17

**Authors:** Qian Zhang

**Affiliations:** Beijing Normal University, Beijing, Beijing, China (People's Republic)

## Abstract

Aging is a global trend and China is no exception. Older people in China mostly rely on their adult children for old-age support. This traditional provision pattern of old-age support, however, is challenged by hundreds of millions of internal migrant workers. They relocate from rural to urban regions for better employment and are no longer able to provide old-age support to their older parents in rural areas. The aim of this study was to determine the impacts of China’s public pension program expansion in rural areas on older people’s expectations for old-age support. Utilizing the natural experiment of program expansion, this study identified an instrumental variable as the county adoption of the pension program. In addition, the study analyzed a nationally representative longitudinal dataset CHARLS with fixed effects model. Results from the statistical model showed that given the participation in the pension program, older adults reported more reliance on pension for old-age support financially and less reliance on children. Heterogeneous effects were found for older adults living together with children and older adults living independently. These important findings suggest that the government partially assumes the responsibility for the old-age support of adult children in the traditional sense. The potential benefits of this study provide a policy implication for developing countries to alleviate old-age support problems and enable internal migration for economic development.

